# 4-(4-Methoxy­phen­yl)-4-methyl-2,6-diphenyl-4*H*-thio­pyran

**DOI:** 10.1107/S1600536809005935

**Published:** 2009-02-25

**Authors:** Hossein Rahmani, Hooshang Pirelahi, Seik Weng Ng

**Affiliations:** a Institute of Chemical Industries, Iranian Research Organization for Science and Technology, PO Box 15815-358, Tehran, Iran; b Department of Chemistry, College of Science, University of Tehran, PO Box 13145-143, Tehran, Iran; c Department of Chemistry, University of Malaya, 50603 Kuala Lumpur, Malaysia

## Abstract

The asymmetric unit of the title compound, C_25_H_22_OS, comprises two similar 4-(4-methoxy­phen­yl)-4-methyl-2,6-diphenyl-4*H*-thio­pyran mol­ecules. In each, the six-membered thio­pyran ring adopts a planar conformation (r.m.s. deviation of 0.041Å for the ring in one mol­ecule and 0.008 Å in the other). The methoxy­phenyl substituent is in a pseudo-axial position. The crystal studied is an inversion twin, with a domain ratio of 0.39 (6).

## Related literature

For the background to 4-alkyl-2,4,6-triaryl-4*H*-thio­pyrans, see: Rahmani *et al.* (2009 ▶). For the general synthesis from a Grignard reaction, see: Suld & Price (1962 ▶).
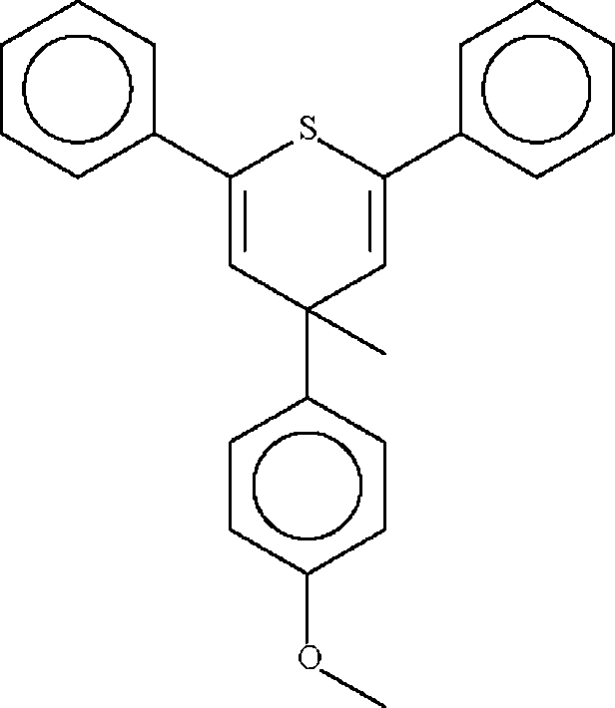


## Experimental

### 

#### Crystal data

C_25_H_22_OS
*M* *_r_* = 370.49
Orthorhombic, 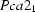

*a* = 14.1567 (2) Å
*b* = 7.6138 (1) Å
*c* = 36.1457 (6) Å
*V* = 3896.0 (1) Å^3^
*Z* = 8
Mo *K*α radiation
μ = 0.18 mm^−1^
*T* = 115 K
0.30 × 0.20 × 0.10 mm

#### Data collection

Bruker SMART APEX diffractometer
Absorption correction: multi-scan (*SADABS*; Sheldrick, 1996 ▶) *T*_min_ = 0.919, *T*_max_ = 0.982
35118 measured reflections
8811 independent reflections
7499 reflections with *I* > 2˘*I*)
*R*_int_ = 0.048

#### Refinement

*R*[*F* ^2^ > 2σ(*F* ^2^)] = 0.040
*wR*(*F* ^2^) = 0.106
*S* = 1.04
8811 reflections
492 parameters
1 restraint
H-atom parameters constrained
Δρ_max_ = 0.27 e Å^−3^
Δρ_min_ = −0.26 e Å^−3^
Absolute structure: Flack (1983 ▶), 4271 Friedel pairs
Flack parameter: 0.39 (6)

### 

Data collection: *APEX2* (Bruker, 2008 ▶); cell refinement: *SAINT* (Bruker, 2008 ▶); data reduction: *SAINT*; program(s) used to solve structure: *SHELXS97* (Sheldrick, 2008 ▶); program(s) used to refine structure: *SHELXL97* (Sheldrick, 2008 ▶); molecular graphics: *X-SEED* (Barbour, 2001 ▶); software used to prepare material for publication: *publCIF* (Westrip, 2009 ▶).

## Supplementary Material

Crystal structure: contains datablocks global, I. DOI: 10.1107/S1600536809005935/sj2580sup1.cif

Structure factors: contains datablocks I. DOI: 10.1107/S1600536809005935/sj2580Isup2.hkl

Additional supplementary materials:  crystallographic information; 3D view; checkCIF report

## Figures and Tables

**Fig. 1.**
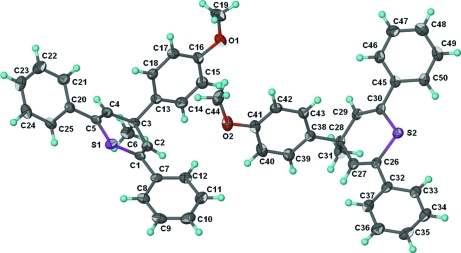
Thermal ellisoid plot (Barbour, 2001) of C24H19BrS; probability levels are set at 70% and H-atoms are drawn as spheres of arbitrary radius.

## supplementary crystallographic information

### Experimental

The compound was synthesized by the reaction of methyl magnesium bromide and 4-(4-anisyl)-2,6-diphenyl thiopyrylium perchlorate in dry ether under an argon atmosphere according to a reported method (Suld & Price, 1962). The product was isolated by TLC on neutral alumina (petroleum ether 40–60 °C) and purified by recrystalization from ethanol.

### Refinement

Carbon-bound H-atoms were placed in calculated positions (C–H 0.95 to 0.98 Å) and were included in the refinement in the riding model approximation, with *U*(H) set to 1.2 to 1.5*U*(C).

The final difference Fourier map had a large peak/deep hole in the vicinity of the bromine. The crystal studied is an inversion, with a twin component of 0.39 (6).

### Crystal data

**Table:**

C_25_H_22_OS	*F*(000) = 1568
*M**_r_* = 370.49	*D*_x_ = 1.263 Mg m^−^^3^
Orthorhombic, *P**c**a*2_1_	Mo *K*α radiation, λ = 0.71073 Å
Hall symbol: P 2c -2ac	Cell parameters from 6600 reflections
*a* = 14.1567 (2) Å	θ = 2.7–25.9°
*b* = 7.6138 (1) Å	µ = 0.18 mm^−^^1^
*c* = 36.1457 (6) Å	*T* = 115 K
*V* = 3896.0 (1) Å^3^	Prism, pale yellow
*Z* = 8	0.30 × 0.20 × 0.10 mm

### Data collection

**Table:**

Bruker SMART APEX diffractometer	8811 independent reflections
Radiation source: fine-focus sealed tube	7499 reflections with *I* > 2˘*I*)
graphite	*R*_int_ = 0.048
ω scans	θ_max_ = 27.5°, θ_min_ = 1.1°
Absorption correction: multi-scan (*SADABS*; Sheldrick, 1996)	*h* = −18→18
*T*_min_ = 0.919, *T*_max_ = 0.982	*k* = −9→9
35118 measured reflections	*l* = −46→46

### Refinement

**Table:**

Refinement on *F*^2^	Secondary atom site location: difference Fourier map
Least-squares matrix: full	Hydrogen site location: inferred from neighbouring sites
*R*[*F*^2^ > 2σ(*F*^2^)] = 0.040	H-atom parameters constrained
*wR*(*F*^2^) = 0.106	*w* = 1/[σ^2^(*F*_o_^2^) + (0.0584*P*)^2^ + 0.3658*P*] where *P* = (*F*_o_^2^ + 2*F*_c_^2^)/3
*S* = 1.04	(Δ/σ)_max_ = 0.001
8811 reflections	Δρ_max_ = 0.27 e Å^−^^3^
492 parameters	Δρ_min_ = −0.25 e Å^−^^3^
1 restraint	Absolute structure: Flack (1983), 4271 Friedel pairs
Primary atom site location: structure-invariant direct methods	Flack parameter: 0.39 (6)

### Fractional atomic coordinates and isotropic or equivalent isotropic displacement parameters (Å^2^)

**Table:**

	*x*	*y*	*z*	*U*_iso_*/*U*_eq_	
S1	0.74555 (4)	0.75526 (7)	0.500000 (18)	0.02190 (12)	
S2	0.48342 (4)	0.25554 (7)	0.150501 (17)	0.02188 (12)	
O1	0.93417 (12)	0.9368 (3)	0.30170 (5)	0.0335 (4)	
O2	0.68606 (14)	0.6061 (3)	0.34833 (5)	0.0456 (5)	
C1	0.66819 (14)	0.8115 (3)	0.46382 (6)	0.0190 (5)	
C2	0.67887 (15)	0.9498 (3)	0.44191 (7)	0.0213 (5)	
H2	0.6296	0.9691	0.4245	0.026*	
C3	0.75898 (15)	1.0807 (3)	0.44093 (6)	0.0198 (5)	
C4	0.82518 (15)	1.0587 (3)	0.47343 (6)	0.0206 (5)	
H4	0.8723	1.1465	0.4761	0.025*	
C5	0.82586 (14)	0.9317 (3)	0.49885 (6)	0.0188 (4)	
C6	0.71782 (18)	1.2682 (3)	0.44237 (7)	0.0274 (5)	
H6A	0.6749	1.2856	0.4214	0.041*	
H6B	0.7693	1.3539	0.4410	0.041*	
H6C	0.6831	1.2841	0.4656	0.041*	
C7	0.58948 (14)	0.6828 (3)	0.45931 (6)	0.0196 (5)	
C8	0.54589 (16)	0.6034 (3)	0.48994 (7)	0.0234 (5)	
H8	0.5661	0.6320	0.5143	0.028*	
C9	0.47374 (17)	0.4837 (3)	0.48484 (8)	0.0271 (6)	
H9	0.4449	0.4303	0.5057	0.033*	
C10	0.44330 (17)	0.4411 (3)	0.44995 (8)	0.0304 (6)	
H10	0.3931	0.3597	0.4467	0.036*	
C11	0.48646 (17)	0.5181 (4)	0.41938 (8)	0.0305 (6)	
H11	0.4661	0.4880	0.3952	0.037*	
C12	0.55884 (16)	0.6381 (3)	0.42407 (7)	0.0252 (5)	
H12	0.5878	0.6902	0.4030	0.030*	
C13	0.81171 (15)	1.0511 (3)	0.40405 (6)	0.0190 (5)	
C14	0.76714 (17)	1.0842 (3)	0.37024 (7)	0.0279 (5)	
H14	0.7055	1.1336	0.3703	0.033*	
C15	0.80995 (17)	1.0473 (3)	0.33695 (7)	0.0298 (5)	
H15	0.7780	1.0718	0.3144	0.036*	
C16	0.89975 (17)	0.9743 (3)	0.33624 (7)	0.0252 (5)	
C17	0.94639 (16)	0.9416 (3)	0.36914 (7)	0.0248 (5)	
H17	1.0082	0.8928	0.3690	0.030*	
C18	0.90181 (16)	0.9809 (3)	0.40252 (7)	0.0226 (5)	
H18	0.9344	0.9587	0.4250	0.027*	
C19	1.0242 (2)	0.8520 (4)	0.29948 (8)	0.0426 (7)	
H19A	1.0396	0.8288	0.2735	0.064*	
H19B	1.0220	0.7408	0.3131	0.064*	
H19C	1.0726	0.9282	0.3103	0.064*	
C20	0.89736 (15)	0.9209 (3)	0.52889 (6)	0.0195 (5)	
C21	0.99097 (17)	0.9677 (3)	0.52196 (7)	0.0244 (5)	
H21	1.0085	1.0078	0.4980	0.029*	
C22	1.05849 (18)	0.9563 (3)	0.54949 (8)	0.0314 (6)	
H22	1.1219	0.9892	0.5444	0.038*	
C23	1.03454 (18)	0.8975 (3)	0.58441 (7)	0.0283 (5)	
H23	1.0810	0.8907	0.6033	0.034*	
C24	0.94234 (18)	0.8488 (3)	0.59161 (7)	0.0284 (5)	
H24	0.9256	0.8073	0.6155	0.034*	
C25	0.87397 (16)	0.8599 (3)	0.56422 (6)	0.0242 (5)	
H25	0.8108	0.8259	0.5695	0.029*	
C26	0.40910 (15)	0.3075 (3)	0.18818 (6)	0.0188 (5)	
C27	0.42127 (16)	0.4450 (3)	0.21015 (7)	0.0219 (5)	
H27	0.3762	0.4574	0.2294	0.026*	
C28	0.49713 (16)	0.5847 (3)	0.20873 (6)	0.0205 (5)	
C29	0.56616 (16)	0.5548 (3)	0.17759 (6)	0.0205 (5)	
H29	0.6165	0.6368	0.1760	0.025*	
C30	0.56606 (14)	0.4290 (3)	0.15210 (6)	0.0182 (4)	
C31	0.44897 (18)	0.7633 (3)	0.20161 (7)	0.0284 (5)	
H31A	0.4011	0.7845	0.2207	0.043*	
H31B	0.4188	0.7619	0.1772	0.043*	
H31C	0.4964	0.8570	0.2024	0.043*	
C32	0.33001 (15)	0.1805 (3)	0.19254 (6)	0.0207 (5)	
C33	0.28735 (16)	0.1024 (3)	0.16188 (7)	0.0232 (5)	
H33	0.3098	0.1288	0.1377	0.028*	
C34	0.21227 (17)	−0.0137 (3)	0.16624 (7)	0.0266 (6)	
H34	0.1841	−0.0667	0.1452	0.032*	
C35	0.17863 (17)	−0.0519 (3)	0.20120 (8)	0.0299 (6)	
H35	0.1272	−0.1306	0.2042	0.036*	
C36	0.22024 (18)	0.0251 (4)	0.23171 (8)	0.0312 (6)	
H36	0.1968	−0.0001	0.2558	0.037*	
C37	0.29591 (17)	0.1387 (3)	0.22755 (7)	0.0266 (5)	
H37	0.3248	0.1886	0.2488	0.032*	
C38	0.54983 (16)	0.5857 (3)	0.24607 (6)	0.0200 (5)	
C39	0.50873 (17)	0.6610 (4)	0.27743 (7)	0.0308 (6)	
H39	0.4476	0.7118	0.2757	0.037*	
C40	0.55541 (19)	0.6629 (4)	0.31089 (7)	0.0364 (6)	
H40	0.5258	0.7140	0.3319	0.044*	
C41	0.64487 (17)	0.5912 (3)	0.31433 (7)	0.0292 (5)	
C42	0.68627 (16)	0.5117 (3)	0.28390 (7)	0.0252 (5)	
H42	0.7467	0.4585	0.2860	0.030*	
C43	0.63812 (16)	0.5107 (3)	0.25018 (7)	0.0220 (5)	
H43	0.6670	0.4567	0.2294	0.026*	
C44	0.78270 (19)	0.5650 (4)	0.35186 (7)	0.0374 (6)	
H44A	0.8047	0.5987	0.3766	0.056*	
H44B	0.8188	0.6294	0.3331	0.056*	
H44C	0.7918	0.4385	0.3484	0.056*	
C45	0.63793 (16)	0.4168 (3)	0.12220 (6)	0.0202 (5)	
C46	0.73151 (17)	0.4634 (3)	0.12934 (7)	0.0244 (5)	
H46	0.7490	0.5021	0.1534	0.029*	
C47	0.79934 (18)	0.4539 (4)	0.10169 (7)	0.0304 (6)	
H47	0.8627	0.4869	0.1069	0.036*	
C48	0.77508 (18)	0.3966 (3)	0.06668 (7)	0.0297 (6)	
H48	0.8215	0.3907	0.0477	0.036*	
C49	0.68272 (18)	0.3478 (3)	0.05938 (7)	0.0285 (5)	
H49	0.6658	0.3077	0.0354	0.034*	
C50	0.61485 (17)	0.3571 (3)	0.08691 (6)	0.0241 (5)	
H50	0.5518	0.3224	0.0817	0.029*	

### Atomic displacement parameters (Å^2^)

**Table:**

	*U*^11^	*U*^22^	*U*^33^	*U*^12^	*U*^13^	*U*^23^
S1	0.0191 (2)	0.0190 (3)	0.0276 (3)	−0.0019 (2)	−0.0007 (2)	0.0044 (2)
S2	0.0198 (2)	0.0198 (3)	0.0260 (3)	−0.0011 (2)	−0.0004 (2)	−0.0050 (2)
O1	0.0322 (9)	0.0453 (11)	0.0229 (9)	−0.0017 (8)	0.0060 (8)	−0.0056 (8)
O2	0.0370 (11)	0.0760 (16)	0.0238 (10)	0.0019 (10)	−0.0068 (8)	−0.0092 (9)
C1	0.0156 (10)	0.0206 (11)	0.0208 (11)	0.0011 (9)	0.0047 (9)	−0.0027 (9)
C2	0.0184 (10)	0.0237 (12)	0.0219 (12)	0.0008 (9)	0.0012 (9)	0.0003 (10)
C3	0.0180 (10)	0.0183 (11)	0.0233 (12)	−0.0007 (8)	0.0035 (9)	0.0005 (9)
C4	0.0215 (11)	0.0188 (12)	0.0214 (12)	−0.0015 (9)	0.0031 (9)	−0.0009 (9)
C5	0.0180 (10)	0.0186 (11)	0.0197 (11)	0.0003 (8)	0.0056 (9)	−0.0034 (9)
C6	0.0264 (12)	0.0222 (12)	0.0337 (14)	0.0043 (9)	0.0030 (11)	−0.0009 (10)
C7	0.0149 (10)	0.0173 (11)	0.0267 (12)	0.0031 (8)	0.0031 (9)	−0.0004 (9)
C8	0.0216 (11)	0.0216 (12)	0.0271 (13)	0.0030 (9)	0.0048 (10)	0.0009 (9)
C9	0.0249 (12)	0.0214 (13)	0.0350 (15)	0.0005 (9)	0.0101 (11)	0.0043 (10)
C10	0.0235 (12)	0.0225 (13)	0.0451 (17)	−0.0063 (10)	0.0025 (11)	−0.0030 (11)
C11	0.0257 (12)	0.0321 (14)	0.0338 (15)	−0.0052 (11)	−0.0018 (11)	−0.0032 (11)
C12	0.0232 (12)	0.0260 (13)	0.0265 (13)	−0.0012 (10)	0.0031 (10)	0.0025 (10)
C13	0.0195 (11)	0.0188 (11)	0.0189 (11)	−0.0030 (9)	0.0028 (8)	0.0030 (9)
C14	0.0216 (11)	0.0339 (14)	0.0281 (13)	0.0048 (10)	−0.0008 (10)	0.0029 (11)
C15	0.0272 (12)	0.0404 (15)	0.0219 (12)	−0.0008 (11)	−0.0014 (10)	0.0065 (11)
C16	0.0294 (12)	0.0261 (12)	0.0200 (11)	−0.0078 (10)	0.0042 (10)	−0.0044 (10)
C17	0.0202 (11)	0.0281 (13)	0.0259 (13)	0.0024 (9)	0.0016 (10)	−0.0008 (11)
C18	0.0206 (11)	0.0262 (13)	0.0209 (13)	−0.0029 (9)	−0.0024 (9)	0.0028 (9)
C19	0.0370 (15)	0.0504 (19)	0.0404 (16)	0.0009 (13)	0.0151 (13)	−0.0112 (14)
C20	0.0234 (11)	0.0150 (11)	0.0200 (11)	0.0001 (9)	0.0019 (9)	−0.0028 (9)
C21	0.0231 (11)	0.0241 (11)	0.0260 (13)	−0.0042 (10)	0.0012 (10)	0.0031 (10)
C22	0.0269 (13)	0.0298 (14)	0.0373 (15)	−0.0076 (11)	−0.0041 (11)	0.0039 (12)
C23	0.0354 (13)	0.0214 (12)	0.0280 (13)	0.0005 (10)	−0.0106 (11)	−0.0005 (10)
C24	0.0414 (15)	0.0255 (13)	0.0182 (12)	0.0045 (11)	0.0002 (11)	0.0013 (10)
C25	0.0262 (12)	0.0243 (13)	0.0223 (12)	0.0006 (9)	0.0051 (10)	−0.0007 (9)
C26	0.0173 (11)	0.0179 (11)	0.0211 (11)	0.0027 (8)	−0.0044 (8)	−0.0006 (9)
C27	0.0216 (11)	0.0218 (12)	0.0224 (12)	0.0019 (9)	−0.0024 (9)	−0.0013 (10)
C28	0.0229 (11)	0.0196 (11)	0.0189 (11)	−0.0016 (9)	−0.0022 (9)	−0.0033 (9)
C29	0.0224 (11)	0.0200 (12)	0.0191 (12)	−0.0043 (9)	−0.0038 (9)	0.0043 (9)
C30	0.0195 (10)	0.0181 (10)	0.0170 (11)	0.0001 (8)	−0.0043 (9)	0.0031 (9)
C31	0.0303 (13)	0.0225 (13)	0.0324 (14)	0.0029 (10)	−0.0022 (11)	0.0004 (10)
C32	0.0176 (11)	0.0177 (11)	0.0267 (12)	0.0024 (8)	−0.0045 (9)	−0.0010 (9)
C33	0.0223 (11)	0.0206 (12)	0.0266 (13)	0.0004 (9)	−0.0035 (10)	−0.0003 (9)
C34	0.0258 (12)	0.0190 (12)	0.0350 (15)	−0.0029 (10)	−0.0075 (11)	−0.0029 (10)
C35	0.0225 (12)	0.0252 (13)	0.0420 (16)	−0.0041 (10)	−0.0053 (11)	0.0055 (11)
C36	0.0252 (13)	0.0359 (14)	0.0324 (15)	−0.0058 (11)	−0.0006 (11)	0.0044 (11)
C37	0.0242 (12)	0.0289 (13)	0.0266 (12)	−0.0009 (10)	−0.0045 (10)	−0.0007 (10)
C38	0.0227 (11)	0.0149 (10)	0.0223 (12)	−0.0030 (8)	0.0008 (9)	−0.0010 (9)
C39	0.0251 (12)	0.0380 (15)	0.0294 (13)	0.0074 (11)	−0.0023 (10)	−0.0100 (12)
C40	0.0354 (14)	0.0490 (17)	0.0246 (13)	0.0040 (12)	0.0019 (11)	−0.0118 (12)
C41	0.0304 (13)	0.0367 (14)	0.0206 (12)	−0.0034 (11)	−0.0006 (10)	−0.0021 (10)
C42	0.0191 (11)	0.0310 (13)	0.0254 (12)	0.0006 (9)	0.0008 (9)	0.0029 (10)
C43	0.0239 (11)	0.0218 (12)	0.0204 (12)	−0.0005 (9)	0.0018 (10)	−0.0018 (10)
C44	0.0370 (15)	0.0446 (17)	0.0305 (14)	−0.0059 (12)	−0.0127 (12)	0.0014 (12)
C45	0.0257 (11)	0.0155 (11)	0.0196 (11)	0.0007 (9)	−0.0016 (9)	0.0027 (9)
C46	0.0274 (12)	0.0227 (12)	0.0230 (13)	−0.0034 (10)	−0.0026 (10)	−0.0021 (10)
C47	0.0248 (12)	0.0324 (14)	0.0338 (15)	−0.0054 (11)	0.0032 (11)	−0.0012 (11)
C48	0.0343 (14)	0.0244 (13)	0.0303 (14)	0.0001 (11)	0.0065 (12)	0.0008 (10)
C49	0.0400 (15)	0.0241 (13)	0.0214 (12)	0.0008 (11)	0.0008 (11)	−0.0018 (10)
C50	0.0277 (12)	0.0221 (12)	0.0226 (12)	−0.0011 (9)	−0.0042 (10)	0.0006 (9)

### Geometric parameters (Å, °)

**Table:**

S1—C1	1.759 (2)	C23—H23	0.9500
S1—C5	1.761 (2)	C24—C25	1.387 (3)
S2—C30	1.766 (2)	C24—H24	0.9500
S2—C26	1.766 (2)	C25—H25	0.9500
O1—C16	1.370 (3)	C26—C27	1.325 (3)
O1—C19	1.431 (3)	C26—C32	1.488 (3)
O2—C41	1.365 (3)	C27—C28	1.513 (3)
O2—C44	1.409 (3)	C27—H27	0.9500
C1—C2	1.326 (3)	C28—C29	1.508 (3)
C1—C7	1.493 (3)	C28—C38	1.542 (3)
C2—C3	1.510 (3)	C28—C31	1.543 (3)
C2—H2	0.9500	C29—C30	1.328 (3)
C3—C4	1.512 (3)	C29—H29	0.9500
C3—C6	1.542 (3)	C30—C45	1.487 (3)
C3—C13	1.544 (3)	C31—H31A	0.9800
C4—C5	1.334 (3)	C31—H31B	0.9800
C4—H4	0.9500	C31—H31C	0.9800
C5—C20	1.487 (3)	C32—C37	1.391 (3)
C6—H6A	0.9800	C32—C33	1.395 (3)
C6—H6B	0.9800	C33—C34	1.391 (3)
C6—H6C	0.9800	C33—H33	0.9500
C7—C12	1.388 (3)	C34—C35	1.381 (4)
C7—C8	1.405 (3)	C34—H34	0.9500
C8—C9	1.381 (3)	C35—C36	1.381 (4)
C8—H8	0.9500	C35—H35	0.9500
C9—C10	1.372 (4)	C36—C37	1.385 (3)
C9—H9	0.9500	C36—H36	0.9500
C10—C11	1.392 (4)	C37—H37	0.9500
C10—H10	0.9500	C38—C43	1.382 (3)
C11—C12	1.383 (3)	C38—C39	1.397 (3)
C11—H11	0.9500	C39—C40	1.378 (4)
C12—H12	0.9500	C39—H39	0.9500
C13—C18	1.384 (3)	C40—C41	1.385 (4)
C13—C14	1.398 (3)	C40—H40	0.9500
C14—C15	1.376 (3)	C41—C42	1.385 (3)
C14—H14	0.9500	C42—C43	1.397 (3)
C15—C16	1.388 (3)	C42—H42	0.9500
C15—H15	0.9500	C43—H43	0.9500
C16—C17	1.383 (3)	C44—H44A	0.9800
C17—C18	1.394 (3)	C44—H44B	0.9800
C17—H17	0.9500	C44—H44C	0.9800
C18—H18	0.9500	C45—C50	1.393 (3)
C19—H19A	0.9800	C45—C46	1.396 (3)
C19—H19B	0.9800	C46—C47	1.388 (4)
C19—H19C	0.9800	C46—H46	0.9500
C20—C21	1.395 (3)	C47—C48	1.382 (4)
C20—C25	1.398 (3)	C47—H47	0.9500
C21—C22	1.383 (4)	C48—C49	1.385 (3)
C21—H21	0.9500	C48—H48	0.9500
C22—C23	1.381 (4)	C49—C50	1.385 (3)
C22—H22	0.9500	C49—H49	0.9500
C23—C24	1.382 (4)	C50—H50	0.9500
			
C1—S1—C5	101.46 (11)	C24—C25—H25	119.8
C30—S2—C26	101.64 (11)	C20—C25—H25	119.8
C16—O1—C19	117.5 (2)	C27—C26—C32	123.2 (2)
C41—O2—C44	118.5 (2)	C27—C26—S2	124.19 (19)
C2—C1—C7	122.7 (2)	C32—C26—S2	112.61 (16)
C2—C1—S1	124.50 (18)	C26—C27—C28	128.9 (2)
C7—C1—S1	112.72 (16)	C26—C27—H27	115.6
C1—C2—C3	128.6 (2)	C28—C27—H27	115.6
C1—C2—H2	115.7	C29—C28—C27	112.27 (19)
C3—C2—H2	115.7	C29—C28—C38	109.90 (18)
C2—C3—C4	111.95 (19)	C27—C28—C38	108.47 (18)
C2—C3—C6	109.04 (19)	C29—C28—C31	107.18 (19)
C4—C3—C6	108.10 (19)	C27—C28—C31	108.18 (19)
C2—C3—C13	106.66 (18)	C38—C28—C31	110.84 (18)
C4—C3—C13	110.78 (18)	C30—C29—C28	128.8 (2)
C6—C3—C13	110.31 (19)	C30—C29—H29	115.6
C5—C4—C3	128.3 (2)	C28—C29—H29	115.6
C5—C4—H4	115.8	C29—C30—C45	123.3 (2)
C3—C4—H4	115.8	C29—C30—S2	124.23 (18)
C4—C5—C20	123.3 (2)	C45—C30—S2	112.48 (16)
C4—C5—S1	124.38 (18)	C28—C31—H31A	109.5
C20—C5—S1	112.33 (16)	C28—C31—H31B	109.5
C3—C6—H6A	109.5	H31A—C31—H31B	109.5
C3—C6—H6B	109.5	C28—C31—H31C	109.5
H6A—C6—H6B	109.5	H31A—C31—H31C	109.5
C3—C6—H6C	109.5	H31B—C31—H31C	109.5
H6A—C6—H6C	109.5	C37—C32—C33	118.3 (2)
H6B—C6—H6C	109.5	C37—C32—C26	120.4 (2)
C12—C7—C8	118.7 (2)	C33—C32—C26	121.2 (2)
C12—C7—C1	119.7 (2)	C34—C33—C32	120.8 (2)
C8—C7—C1	121.6 (2)	C34—C33—H33	119.6
C9—C8—C7	120.3 (2)	C32—C33—H33	119.6
C9—C8—H8	119.9	C35—C34—C33	120.1 (2)
C7—C8—H8	119.9	C35—C34—H34	120.0
C10—C9—C8	120.7 (2)	C33—C34—H34	120.0
C10—C9—H9	119.6	C36—C35—C34	119.6 (2)
C8—C9—H9	119.6	C36—C35—H35	120.2
C9—C10—C11	119.5 (2)	C34—C35—H35	120.2
C9—C10—H10	120.2	C35—C36—C37	120.5 (3)
C11—C10—H10	120.2	C35—C36—H36	119.7
C12—C11—C10	120.4 (3)	C37—C36—H36	119.7
C12—C11—H11	119.8	C36—C37—C32	120.7 (2)
C10—C11—H11	119.8	C36—C37—H37	119.7
C11—C12—C7	120.4 (2)	C32—C37—H37	119.7
C11—C12—H12	119.8	C43—C38—C39	117.3 (2)
C7—C12—H12	119.8	C43—C38—C28	122.0 (2)
C18—C13—C14	116.8 (2)	C39—C38—C28	120.7 (2)
C18—C13—C3	122.4 (2)	C40—C39—C38	121.1 (2)
C14—C13—C3	120.7 (2)	C40—C39—H39	119.4
C15—C14—C13	121.9 (2)	C38—C39—H39	119.4
C15—C14—H14	119.0	C39—C40—C41	120.9 (2)
C13—C14—H14	119.0	C39—C40—H40	119.6
C14—C15—C16	120.1 (2)	C41—C40—H40	119.6
C14—C15—H15	120.0	O2—C41—C40	116.0 (2)
C16—C15—H15	120.0	O2—C41—C42	124.8 (2)
O1—C16—C17	125.2 (2)	C40—C41—C42	119.2 (2)
O1—C16—C15	115.2 (2)	C41—C42—C43	119.3 (2)
C17—C16—C15	119.6 (2)	C41—C42—H42	120.4
C16—C17—C18	119.3 (2)	C43—C42—H42	120.4
C16—C17—H17	120.3	C38—C43—C42	122.2 (2)
C18—C17—H17	120.3	C38—C43—H43	118.9
C13—C18—C17	122.3 (2)	C42—C43—H43	118.9
C13—C18—H18	118.8	O2—C44—H44A	109.5
C17—C18—H18	118.8	O2—C44—H44B	109.5
O1—C19—H19A	109.5	H44A—C44—H44B	109.5
O1—C19—H19B	109.5	O2—C44—H44C	109.5
H19A—C19—H19B	109.5	H44A—C44—H44C	109.5
O1—C19—H19C	109.5	H44B—C44—H44C	109.5
H19A—C19—H19C	109.5	C50—C45—C46	118.4 (2)
H19B—C19—H19C	109.5	C50—C45—C30	121.7 (2)
C21—C20—C25	118.3 (2)	C46—C45—C30	119.9 (2)
C21—C20—C5	120.1 (2)	C47—C46—C45	120.7 (2)
C25—C20—C5	121.6 (2)	C47—C46—H46	119.7
C22—C21—C20	120.8 (2)	C45—C46—H46	119.7
C22—C21—H21	119.6	C48—C47—C46	120.3 (2)
C20—C21—H21	119.6	C48—C47—H47	119.9
C23—C22—C21	120.6 (2)	C46—C47—H47	119.9
C23—C22—H22	119.7	C47—C48—C49	119.6 (2)
C21—C22—H22	119.7	C47—C48—H48	120.2
C22—C23—C24	119.4 (2)	C49—C48—H48	120.2
C22—C23—H23	120.3	C48—C49—C50	120.3 (2)
C24—C23—H23	120.3	C48—C49—H49	119.9
C23—C24—C25	120.5 (2)	C50—C49—H49	119.9
C23—C24—H24	119.7	C49—C50—C45	120.8 (2)
C25—C24—H24	119.7	C49—C50—H50	119.6
C24—C25—C20	120.5 (2)	C45—C50—H50	119.6
			
C5—S1—C1—C2	3.5 (2)	C30—S2—C26—C27	−0.8 (2)
C5—S1—C1—C7	−178.39 (15)	C30—S2—C26—C32	178.67 (15)
C7—C1—C2—C3	−174.0 (2)	C32—C26—C27—C28	−179.3 (2)
S1—C1—C2—C3	4.0 (4)	S2—C26—C27—C28	0.2 (4)
C1—C2—C3—C4	−9.7 (3)	C26—C27—C28—C29	−0.5 (3)
C1—C2—C3—C6	−129.3 (3)	C26—C27—C28—C38	−122.1 (3)
C1—C2—C3—C13	111.6 (3)	C26—C27—C28—C31	117.6 (3)
C2—C3—C4—C5	7.7 (3)	C27—C28—C29—C30	2.0 (3)
C6—C3—C4—C5	127.8 (3)	C38—C28—C29—C30	122.8 (2)
C13—C3—C4—C5	−111.2 (3)	C31—C28—C29—C30	−116.6 (3)
C3—C4—C5—C20	177.6 (2)	C28—C29—C30—C45	178.7 (2)
C3—C4—C5—S1	−0.3 (3)	C28—C29—C30—S2	−3.1 (3)
C1—S1—C5—C4	−5.2 (2)	C26—S2—C30—C29	2.2 (2)
C1—S1—C5—C20	176.71 (15)	C26—S2—C30—C45	−179.44 (15)
C2—C1—C7—C12	36.8 (3)	C27—C26—C32—C37	−34.2 (3)
S1—C1—C7—C12	−141.36 (19)	S2—C26—C32—C37	146.29 (19)
C2—C1—C7—C8	−144.0 (2)	C27—C26—C32—C33	144.9 (2)
S1—C1—C7—C8	37.8 (3)	S2—C26—C32—C33	−34.6 (3)
C12—C7—C8—C9	−0.3 (3)	C37—C32—C33—C34	0.3 (3)
C1—C7—C8—C9	−179.5 (2)	C26—C32—C33—C34	−178.8 (2)
C7—C8—C9—C10	−0.3 (4)	C32—C33—C34—C35	0.5 (4)
C8—C9—C10—C11	0.7 (4)	C33—C34—C35—C36	−0.3 (4)
C9—C10—C11—C12	−0.7 (4)	C34—C35—C36—C37	−0.6 (4)
C10—C11—C12—C7	0.2 (4)	C35—C36—C37—C32	1.5 (4)
C8—C7—C12—C11	0.3 (4)	C33—C32—C37—C36	−1.3 (4)
C1—C7—C12—C11	179.5 (2)	C26—C32—C37—C36	177.8 (2)
C2—C3—C13—C18	−110.6 (2)	C29—C28—C38—C43	−20.2 (3)
C4—C3—C13—C18	11.5 (3)	C27—C28—C38—C43	102.9 (2)
C6—C3—C13—C18	131.1 (2)	C31—C28—C38—C43	−138.4 (2)
C2—C3—C13—C14	65.2 (3)	C29—C28—C38—C39	160.8 (2)
C4—C3—C13—C14	−172.7 (2)	C27—C28—C38—C39	−76.1 (3)
C6—C3—C13—C14	−53.1 (3)	C31—C28—C38—C39	42.5 (3)
C18—C13—C14—C15	0.7 (4)	C43—C38—C39—C40	1.1 (4)
C3—C13—C14—C15	−175.3 (2)	C28—C38—C39—C40	−179.8 (2)
C13—C14—C15—C16	0.3 (4)	C38—C39—C40—C41	0.5 (4)
C19—O1—C16—C17	2.1 (4)	C44—O2—C41—C40	−168.6 (2)
C19—O1—C16—C15	−176.5 (2)	C44—O2—C41—C42	11.0 (4)
C14—C15—C16—O1	177.6 (2)	C39—C40—C41—O2	177.6 (3)
C14—C15—C16—C17	−1.0 (4)	C39—C40—C41—C42	−2.1 (4)
O1—C16—C17—C18	−177.8 (2)	O2—C41—C42—C43	−177.6 (2)
C15—C16—C17—C18	0.7 (3)	C40—C41—C42—C43	2.0 (4)
C14—C13—C18—C17	−1.0 (3)	C39—C38—C43—C42	−1.2 (3)
C3—C13—C18—C17	174.9 (2)	C28—C38—C43—C42	179.8 (2)
C16—C17—C18—C13	0.4 (4)	C41—C42—C43—C38	−0.4 (4)
C4—C5—C20—C21	−37.1 (3)	C29—C30—C45—C50	−144.9 (2)
S1—C5—C20—C21	141.01 (19)	S2—C30—C45—C50	36.7 (3)
C4—C5—C20—C25	144.6 (2)	C29—C30—C45—C46	36.0 (3)
S1—C5—C20—C25	−37.3 (3)	S2—C30—C45—C46	−142.44 (19)
C25—C20—C21—C22	−0.9 (4)	C50—C45—C46—C47	1.3 (4)
C5—C20—C21—C22	−179.2 (2)	C30—C45—C46—C47	−179.6 (2)
C20—C21—C22—C23	0.3 (4)	C45—C46—C47—C48	−0.5 (4)
C21—C22—C23—C24	0.4 (4)	C46—C47—C48—C49	−0.3 (4)
C22—C23—C24—C25	−0.6 (4)	C47—C48—C49—C50	0.3 (4)
C23—C24—C25—C20	−0.1 (4)	C48—C49—C50—C45	0.5 (4)
C21—C20—C25—C24	0.8 (3)	C46—C45—C50—C49	−1.3 (3)
C5—C20—C25—C24	179.1 (2)	C30—C45—C50—C49	179.6 (2)
